# Nurse Staffing in Psychiatric Inpatient Care: A Multicentre Study Using Routine Patient and Structural Data

**DOI:** 10.1155/jonm/5552920

**Published:** 2026-07-30

**Authors:** Michael Ketzer, Beatrice Gehri, Christian G. Huber, André Nienaber, Michael Simon

**Affiliations:** ^1^ Department Public Health, Institute of Nursing Science, University of Basel, Basel, Switzerland, unibas.ch; ^2^ University Psychiatric Clinics (UPK) Basel, University of Basel, Basel, Switzerland, unibas.ch; ^3^ Faculty of Business Management and Social Sciences, Osnabrück University of Applied Sciences, Osnabrück, Germany, hs-osnabrueck.de

**Keywords:** health-of-the-nation outcome scales, inpatients/psychiatric inpatient care, nurse staffing, psychiatric nursing, routine data, workforce planning

## Abstract

**Background:**

Nurse staffing in psychiatric inpatient care is difficult to plan because reliable measures of patient demand are lacking. Existing indicators are not well established across diverse patient groups. The Health of the Nation Outcome Scales (HoNOS), routinely collected in Switzerland, may provide a pragmatic proxy for patient acuity to inform staffing decisions.

**Design and Methods:**

We conducted a multicentre cross‐sectional study (Match^RN^ Psychiatry) in adult, nonforensic psychiatric inpatient units in Switzerland, linking 2022 routine patient data with 2023/2024 unit‐manager reports of typical structural staffing configurations. Patient mental health status and symptom severity were measured using the HoNOS. Associations between nurse staffing hours, HoNOS scores and unit characteristics were analysed using lasso‐selected linear mixed‐effects models. Model estimates were used to describe how RN staffing varied with patient acuity, and model‐predicted staffing was compared with reported staffing to characterise between‐unit variation.

**Results:**

Data from 107 psychiatric inpatient units in 13 hospitals (25,294 patient cases) showed wide variation in unit structures and patient mental health status but relatively little variation in staffing patterns. The median number of beds was 21, and the typical early shift included 2 registered nurses. Regression analyses indicated that higher HoNOS total scores and several single items were significantly associated with increased RN staffing, with the total score showing the strongest effect. A 20% increase in the HoNOS total score corresponded to 48.5 additional registered nurse hours per week or 1.3 full‐time equivalents per year. Comparing predicted with reported staffing, smaller units tended to fall below and larger units above their predicted levels.

**Conclusions:**

Routinely collected clinical data, particularly HoNOS scores, were linked to nurse staffing in psychiatric inpatient care. Patient acuity and unit size were both associated with staffing, indicating that routine data can provide a pragmatic basis for more transparent and sustainable workforce planning.

## 1. Introduction

In the context of an increasing workforce shortage and a growing need for psychiatric services, aligning nurse staffing with patient care demand remains a persistent challenge [1]. Rising mental health care needs and the increased prevalence of mental illness place additional strain on already‐stretched psychiatric care systems [2].

While the importance of appropriate nurse staffing is widely acknowledged, data and methods to guide staffing decisions in mental health settings are limited [3–5]. At the same time, it is recognised that factors beyond staffing, such as leadership, organisational management, reimbursement mechanisms and the presence of research or teaching activities, also shape the quality of care and the availability of staffing resources [6, 7]. Most existing evidence comes from somatic acute care, where associations between staffing and patient outcomes are well documented [8]. In psychiatric inpatient care, the work is less task‐oriented and more relational, shaped by fluctuating patient conditions that are difficult to capture with metrics used in somatic settings [9]. The work environment also differs substantially [10], further complicating the transferability of somatic staffing models.

As with many other key indicators in psychiatry, such as those on treatment outcomes, or service coverage [11], reliable and comparable data on nurse staffing in psychiatric inpatient care remain limited. In the United States, psychiatric units—especially those serving children, adolescents and older adults—tend to have lower proportions of registered nurses (RNs), particularly in for‐profit hospitals [12]. In Switzerland, hospital care is organised at the cantonal level, with no national standards or reporting requirements for nurse staffing, leaving little knowledge about its variation or relationship to patient care demand.

Measuring psychiatric patient needs poses methodological challenges. Unlike in other areas of health care, where various indicators such functional status have been used to approximate care demand, psychiatric outcomes such as aggression, containment or length of stay are highly interdependent, prone to observer bias, and often ambiguous in their clinical meaning [13]. Moreover, the extent to which patient demand translates into staffing requirements is also shaped by structural characteristics of inpatient units, such as ward openness, bed numbers or service configuration. Taken together, these complexities contribute to the lack of indicators that can adequately reflect patient demand and, consequently, inform nurse staffing decisions.

Although mental health‐specific nurse‐sensitive patient‐reported outcomes have been suggested [14], they are still emerging and not widely adopted. Their use usually involves extra documentation effort beyond routine clinical practice. A further challenge lies in the heterogeneity of psychiatric patient populations: major diagnostic groups such as substance use disorders, personality disorders or affective disorders differ substantially in how care needs manifest. Indicators that might be appropriate for one group are not necessarily meaningful for another, making it difficult to define a common set of measures that adequately captures patient care demand across settings. Against this background, routine clinical data offer a pragmatic way to capture patient demand more consistently. It avoids additional administrative burden and enables continuous monitoring [15]. Standardised assessments such as the Health of the Nation Outcome Scales (HoNOS), mandatory at admission and discharge in Switzerland, provide structured information on patient status and are already used for treatment evaluation and quality monitoring [16]; Nationaler Verein für Qualitätsentwicklung in Spitälern und Kliniken [17, 18]. Despite their availability and use in other contexts, their potential for informing staffing decisions in psychiatric inpatient care—alone or in combination with structural unit data—has not been explored in the Swiss context.

To address gaps in understanding the relationship between patient needs and staffing resources in psychiatric inpatient care, this study aimed to (1) describe structural characteristics, patient mental health status and symptom severity and nurse staffing patterns at the unit level in adult, nonforensic psychiatric units in Switzerland; (2) analyse how individual HoNOS items and the HoNOS total score, together with structural unit characteristics, are associated with RN staffing; and (3) estimate staffing levels from these characteristics and compare them with structural staffing patterns.

## 2. Materials and Methods

### 2.1. Study Design and Setting

This study is reported in accordance with the STrengthening the Reporting of OBservational studies in Epidemiology (STROBE) and REporting of studies Conducted using Observational Routinely‐collected Data (RECORD) statements [19, 20]. This study is part of the ‘Matching RN services with changing care demands in psychiatric hospitals’ (Match^RN^ Psychiatry) project [21], a multicentre study in psychiatric hospitals in the German‐speaking part of Switzerland. The project combines cross‐sectional nursing staff and unit surveys with routinely collected time‐series patient data. Its overall aims are to measure and describe the nursing work environment, staffing and quality in psychiatric inpatient care. The Match^RN^ Psychiatry project collected data in two rounds: 2019/2020 and 2023/2024. The present analysis links routine patient data for discharges in 2022, with unit‐manager survey data collected between November 2023 and April 2024. The survey data were used to describe typical structural staffing configurations and unit characteristics, whereas the routine data were used to describe the patient composition of the same units during 2022.

The study region’s responsible ethics commission (Ethics Commission Northwest and Central Switzerland) ruled that the Match^RN^ Psychiatry study is exempt from the Swiss Human Research Act (project ID: Req‐2019‐00589).

### 2.2. Sample and Participants

All psychiatric hospitals represented in the Swiss Psychiatric Nursing Leaders’ Association (VPPS) (*n* = 40) were invited to participate in this study. In the 2023/2024 data‐collection round, 13 hospitals participated with 124 care units. No a priori sample size calculation was performed; instead, this study used a pragmatic sampling approach, inviting all psychiatric hospitals represented by the VPPS. This approach aimed to maximise national coverage of adult nonforensic psychiatric inpatient units. Units were included if they (1) provided 24/7 inpatient care for adults and (2) were classified as nonforensic. All patients hospitalised on these units were included. Participating hospitals were located across the German‐speaking part of Switzerland and included university hospitals, public and private hospitals, with inpatient bed capacity ranging from approximately 70 to 360.

### 2.3. Data Sources and Collection

Two data sources were used. The first was an online survey administered between November 2023 and April 2024 to unit managers or their deputies, collecting information about typical staffing, service provision and unit characteristics. Staffing questions referred to typical staffing patterns at the unit level. The second was patient routine data for all patients discharged between 1 January and 31 December 2022, extracted from the clinical information systems of 13 participating hospitals. Because staffing information was collected after the patient data period, linking survey‐based staffing patterns to patient‐level routine data assumes that unit‐level staffing patterns were sufficiently stable between 2022 and the survey period. Patient data were based on four mandatory reporting files required by the Swiss national quality measurement programme Nationaler Verein für Qualitätsentwicklung in Spitälern und Kliniken [17]. In addition, HoNOS assessment data and information on the patient’s last inpatient unit were obtained separately from each participating hospital to enable unit‐level attribution. Patient data included admission and discharge timestamps, demographic and clinical characteristics, HoNOS assessments, compulsory admission status and use of coercive measures. All data were pseudonymised and transferred securely in accordance with university‐approved data protection procedures.

### 2.4. Data Linkage and Management

**FIGURE 1:**
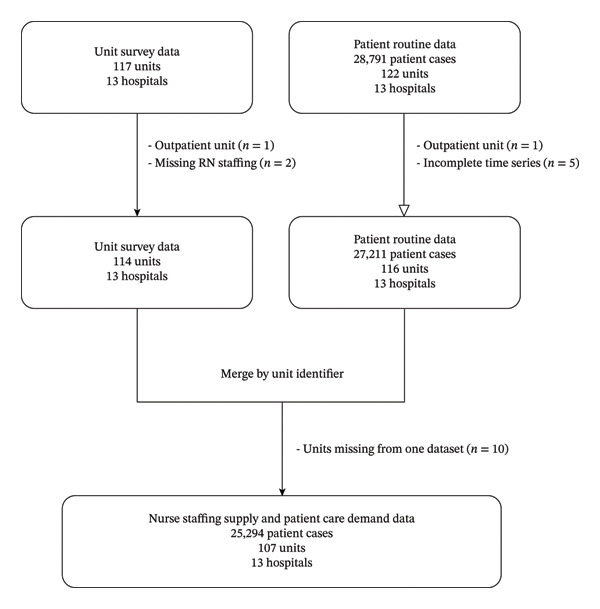
Data flow of merging, in‐/exclusion and final sample size.

Survey and patient data were linked by a unit identifier (Figure 1). The routine dataset was restricted to discharges in 2022; cases discharged after 31 December 2022 were excluded. For staffing data, missing values were replaced with zero where context supported this assumption; otherwise, records were removed. Units with entirely missing staffing data were excluded listwise, and units with incomplete patient data were excluded from analyses relying on those data.

### 2.5. Variables

#### 2.5.1. Patient‐Related Variables

Mental health status and symptom severity were measured with the 12‐item HoNOS [18], which is mandatorily assessed at admission and discharge for national benchmarking in Switzerland [22]. Only admission ratings were used in this study. Each item was dichotomised, with scores of 0–2 classified as nonsevere and scores of 3‐4 classified as severe (Parabiaghi et al., 2014); missing cases and items rated as 9 (not applicable) were coded as 0. Dichotomised item scores were then aggregated to the unit level as the proportion of patients rated severe for each of the 12 items. Aggregation was necessary as staffing data were available on the unit level. In addition, a total HoNOS score was calculated as the sum of all 12 items (range 0–48) per patient and brought to the unit level by computing summary statistics. The dataset further included compulsory admission and coercive measures (both binary at the patient level), which were aggregated to unit‐level proportions. The 12 HoNOS items are reported individually in Tables 1 and 2.

**TABLE 1:** Descriptive statistics of variables included in the analysis.

	Mean (SD)	Median (IQR)	Range
*Dichotomous characteristics*			
Private ownership	14%	—	—
Outpatient service	50%	—	—
Peer support workers	39%	—	—
Unit always open	45%	—	—

*Unit average*			
Capacity utilisation	79% (5%)	80% (76%–82%)	61%–91%
Unit size (number of beds)	21 (5)	21 (18–23)	11–43
Age	50 (16)	42 (39–62)	21–80
Proportion of female patients	50% (13%)	50% (42%–58%)	18%–100%

*Mean proportion of patients with*			
Coercive measures	11% (12%)	7% (1%–17%)	0%–53%
Compulsory admission	25% (21%)	25% (5%–41%)	0%–88%

*Mean of patient*			
Length of stay	31 (13)	29 (20–38)	5–74
HoNOS total score	20 (4)	20 (17–23)	13–32

*HoNOS: Mean proportion of patients with high ratings in*			
(1) Overactive/aggressive/disruptive/agitated behaviour	23% (17%)	19% (9%–34%)	0%–74%
(2) Nonaccidental self‐injury	16% (10%)	14% (9%–21%)	1%–59%
(3) Problem drinking or drug‐taking	30% (24%)	23% (14%–38%)	1%–98%
(4) Cognitive problems	29% (21%)	24% (12%–41%)	1%–91%
(5) Physical illness or disability problems	28% (18%)	24% (16%–36%)	5%–97%
(6) Problems associated with hallucinations/delusions	21% (15%)	21% (7%–30%)	0%–69%
(7) Problems with depressed mood	56% (20%)	55% (41%–72%)	11%–97%
(8) Other mental and behavioural problems	66% (17%)	68% (58%–77%)	25%–97%
(9) Problems with relationships	39% (18%)	38% (26%–48%)	11%–90%
(10) Problems with activities of daily living	44% (22%)	39% (29%–57%)	4%–96%
(11) Problems with housing and living conditions	28% (17%)	26% (15%–37%)	0%–77%
(12) Problems with occupation and activities	39% (19%)	37% (24%–52%)	7%–87%

*Number of registered nurses on unit per shift*			
Day shift (weekday)	2.4 (0.9)	2 (2–3)	1–6
Late shift (weekday)	1.8 (0.6)	2 (1.5–2)	1–3
Night shift (weekday)	1 (0.3)	1 (1–1)	0–2
Day shift (weekend)	1.5 (0.6)	1 (1–2)	1–3
Late shift (weekend)	1.5 (0.6)	1 (1–2)	1–3
Night shift (weekend)	1 (0.4)	1 (1–1)	0–2

*Number of licensed practical nurses on unit per shift*			
Day shift (weekday)	1.1 (0.7)	1 (1–1)	0–4
Late shift (weekday)	0.8 (0.6)	1 (0–1)	0–2
Night shift (weekday)	0.3 (0.5)	0 (0–1)	0–1
Day shift (weekend)	0.8 (0.6)	1 (0–1)	0–3
Late shift (weekend)	0.6 (0.6)	1 (0–1)	0–2
Night shift (weekend)	0.3 (0.5)	0 (0–1)	0–1

**TABLE 2:** Regression estimates and confidence intervals for HoNOS items and total score across three modelling approaches.

HoNOS item/HoNOS total score	Total score model	12 single‐item models	Simultaneous‐item model
HoNOS Total Score (0‐1 scaled)	11.55^∗∗^ (2.95, 20.15)		
(1) Overactive or aggressive or disruptive or agitated behaviour		6.08^∗^ (0.75, 11.41)	
(2) Nonaccidental self‐injury		1.29 (−7.52, 10.10)	−4.00 (−11.98, 3.99)
(3) Problem drinking or drug‐taking		−1.89 (−5.77, 1.99)	−1.14 (−4.52, 2.25)
(4) Cognitive problems		7.10^∗∗^ (2.27, 11.93)	2.51 (−3.33, 8.35)
(5) Physical illness or disability problems		7.94^1^ (−0.17, 16.06)	
(6) Problems associated with hallucinations and/or delusions		2.95 (−2.80, 8.71)	
(7) Problems with depressed mood		3.94^1^ (−0.37, 8.25)	
(8) Other mental and behavioural problems		6.44^∗∗^ (1.86, 11.02)	0.53 (−5.50, 6.56)
(9) Problems with relationships		6.88^∗∗^ (2.78, 10.98)	8.74^∗^ (0.65, 16.82)
(10) Problems with activities of daily living		4.78^∗∗^ (1.15, 8.41)	
(11) Problems with housing and living conditions		3.52 (−1.50, 8.53)	−4.36 (−11.45, 2.73)
(12) Problems with occupation and activities		3.84^∗^ (0.07, 7.61)	

^1^ *p* < 0.1.

^∗^ *p* < 0.05.

^∗∗^ *p* < 0.01.

^∗∗∗^ *p* < 0.001.

#### 2.5.2. Unit and Hospital Characteristics

Unit size was derived from the routine patient data by mapping each patient’s stay to the corresponding unit using admission and discharge timestamps, thereby creating a continuous occupancy profile for 2022. The size was defined as the maximum number of patients simultaneously present on the unit that was sustained for at least 1% of all hourly intervals in the year. Capacity utilisation was calculated as the patient count divided by this bed capacity within a defined timeframe. In Swiss psychiatric care, unit door policies vary: Some units are always open, some are generally open but can be closed temporarily depending on patient needs and some remain closed at all times. For analysis, unit status was reported by managers and dichotomised into ‘always open’ versus ‘partially open or closed’. Within Swiss psychiatric care, some inpatient units also provide outpatient care by reserving places for discharged patients who continue to be followed up and may return for short visits. Peer support workers (PSWs) are trained staff with personal experience as psychiatric patients, providing support to current patients. Whether a unit offered outpatient care or employed PSWs was treated as a binary indicator. Hospital ownership was coded as public or private based on information provided by the study team.

#### 2.5.3. Staffing Variables

RN staffing was reported by unit managers as typical staffing configurations, separately for day, evening and night shifts on both weekdays and weekends, resulting in six distinct shift‐day combinations per unit. RN counts were converted to hours, assuming one RN corresponds to a 9‐hour‐shift, consistent with the Swiss standard (including mandatory breaks). Licensed practical nurse (LPN) staffing was collected in the same way. In Switzerland, most RNs have completed vocational nursing training (Advanced Federal Diploma of Higher Education, *HF*), while a smaller proportion holds a bachelor’s degree in nursing. LPNs complete a 3‐year vocational programme (Federal Diploma of Vocational Education and Training, *EFZ*). Not all hospitals or units employ LPNs, but in Switzerland, inpatient psychiatric care teams often include both RNs, who hold responsibility for care planning and coordination, and LPNs, who provide delegated care within their scope of practice.

### 2.6. Statistical Analysis

Structural, patient and nurse staffing characteristics were summarised at the unit level. Continuous variables were reported as means with standard deviations or medians with interquartile ranges, and categorical variables as counts and percentages.

The dependent variable in all models was RN staffing in hours. LPN staffing was included as one of several covariates, together with patient and unit characteristics. Because the patient data and staffing survey were not collected contemporaneously, model estimates were interpreted as associations between 2022 unit‐level patient composition and subsequently reported typical structural staffing configurations, rather than as causal effects or estimates of staffing responses to short‐term changes in patient acuity. To capture the relationship between patient acuity and structural staffing from different perspectives, we applied three modelling approaches. In the (1) HoNOS total score model, the 12 items were summed, standardised to a 0–1 scale and used as the sole patient‐side predictor, providing an overall measure of patient severity in which each item carries equal weight. The (2) single‐item models entered each HoNOS item separately as the proportion of patients with severe ratings, allowing us to explore the contribution of individual symptom or functioning domains to structural staffing. Finally, the (3) simultaneous‐item model included all 12 item proportions at once, enabling direct comparison of items while accounting for their overlap.

Variable selection was performed separately for each modelling approach using lasso regression. The common candidate set included shift indicators (weekend shift, late shift, night shift), unit and hospital characteristics (unit size, capacity utilisation, private ownership, outpatient service provision, PSW employment and open ward status), patient composition variables (mean age, proportion of female patients, mean length of stay, proportions of compulsory admissions and coercive measures) and LPN staffing. The HoNOS specification differed by the modelling approach: the total score model included the scaled HoNOS total score, the single‐item models included one HoNOS item proportion at a time and the simultaneous‐item model included all 12 HoNOS item proportions. The penalty parameter (λ) was tuned by cross‐validation, and variables retained in at least 67% of bootstrap samples were entered as fixed effects into final linear mixed‐effects models with random intercepts for units to account for clustering (null model ICC = 0.15, 95% CI 0.08–0.22). The final linear mixed‐effects models were estimated without penalisation; the lasso procedure was used only for variable selection. The full candidate set and bootstrap lasso selection outcome are reported in Supporting Table 2.

Model outputs were used for two purposes: first, to estimate associations between changes in patient characteristics, specifically, changes in the proportion of patients with high HoNOS ratings or in the mean HoNOS total score per unit, and RN staffing hours for each of the six shift‐day combinations per unit and when aggregated to the full week and full year; and second, to compare the RN staffing hours estimated by the models with the reported structural staffing hours for the same shifts and aggregated periods, thereby quantifying discrepancies between modelled and reported staffing. The effects of changes in HoNOS were expressed as full‐time equivalent (FTE) RNs, assuming 1 FTE equals 1890 h per year [24].

All statistical analyses were conducted using R, version 4.5.1 for Mac OS [25], using the tidyverse, janitor, glmnet, gt, modelsummary, parameters, rptR and lme4 packages [26–33].

## 3. Results

### 3.1. Descriptive Results

A total of 116 unit managers completed the survey (response rate 94%). Routine patient data were available for 122 units. One unit was excluded after data collection as it did not provide 24/7 inpatient care. Routine patient data from five additional units were excluded due to incomplete or inconsistent coverage of the 2022 reporting period. Analyses were restricted to units with both a completed unit manager survey and a corresponding routine patient dataset. The final sample comprised 107 units from 13 psychiatric hospitals, with linked survey data and patient data for 25,294 cases (Figure 1). Nationally, 77,652 discharges of adult nonforensic inpatient psychiatric treatment cases were recorded in 2020 [22], meaning the final analytic sample corresponds to roughly one‐third of the Swiss national adult psychiatric inpatient population.

Among the 107 units included in the final sample, the mean patient age at the unit level was 45 years (SD 15). The proportion of cases with ‘severe’ ratings varied widely across the 12 HoNOS items ranging from 0% to 74% for item 1 (overactive/aggressive behaviour) and from 11% to 90% for item 9 (problems with relationships). The mean HoNOS total score was 20 (SD 4, possible range 0–48). The proportion of compulsory admissions ranged from 0% to 88% across units, while the proportion of cases with at least one coercive measure ranged from 0% to 53%. The average length of stay was 31 days (SD 13).

Unit characteristics also reflected variation across the sample. Among the units, 47% were consistently open, while the rest was classified as partially open or closed. 52% of units offered outpatient services, and 41% employed PSWs. Units had a median of 21 beds (IQR 18–23). The average capacity utilisation was 75% (SD 5%).

In comparison with patient‐related characteristics, nurse staffing patterns showed less variation across units. The median number of RNs was 2 in the day and late shift during the week, and 1 in the night shift during the week and in all three shifts on the weekend, with interquartile ranges between 1 and 0. LPN staffing was 1 (median) for day and evening shifts on both weekdays and weekends, and 0 for night shifts with IQRs between 0 and 1, indicating generally low LPN staffing levels. Thirteen units did not employ LPNs.

Additional details for patient clinical status, unit characteristics and staffing measures are presented in Table 1.

### 3.2. Inferential Results

Across the three modelling approaches (HoNOS total score model, 12 single‐item models and simultaneous‐single‐item model), lasso regression identified between 14 and 18 covariates. These were retained in the final linear mixed‐effects models, each including a random intercept for unit. The models showed good explanatory power, with conditional *R* ^2^ values ranging from 0.572 to 0.575 across models.

In the HoNOS total score model, the total score was significantly associated with RN staffing (*p* < 0.01). In the single‐item models, six items (1, 4, 8, 9, 10, 12) were significant at *p* < 0.05. In the simultaneous‐item model, six HoNOS items were retained, with one (item 9: problems with relationships) reaching statistical significance at *p* < 0.05.

Among the structural characteristics, unit size was positively associated with RN staffing in all models. As the dependent variable was absolute RN hours rather than a standardised staffing measure such as a patient‐to‐staff ratio, the observed association with unit size reflects that RN staffing was modelled as an absolute volume rather than in relation to patient numbers. The mean capacity utilisation was additionally included to account for differences in occupancy. PSWs and LPN staffing were selected in all models with negative associations, but only the estimate of peer support reached statistical significance in some models, while LPN staffing did not. Length of stay was statistically significant across all models with a negative sign, indicating that longer average stays were linked to fewer RN hours. Full model outputs are provided in Supporting Table 1.

Since the HoNOS total score was also scaled from 0 to 1, the coefficient estimates are directly comparable across models. Among statistically significant estimates, the HoNOS total score in the total score model (modelling approach 1) had the largest coefficient (11.55, 95% CI [2.95, 20.15]) followed by HoNOS item 9 in the simultaneous‐item model (modelling approach 3) (8.74, 95% CI [0.66, 16.82]). In the single‐item models (modelling approach 2), estimates ranged from 6.877, 95% CI [2.78, 10.98] (item 9) to 3.84 95% CI [0.07, 7.61] (item 12) (Table 2).

To illustrate the implications of the model estimates, we calculated the predicted change in RN staffing associated with hypothetical increases in patient acuity, measured either by the proportion of patients with severe ratings on individual HoNOS items or by the scaled HoNOS total score. These calculations were derived from the estimated regression coefficients and served as illustrative model‐based scenarios. Multiplying the respective coefficient by 0.2 yielded the predicted change in RN hours per shift associated with a 20% increase in the acuity measure. The largest estimate was observed for the HoNOS total score, where a 20% increase—equivalent to 9.6 points, about twice the unit‐level standard deviation—was associated with an estimated change of 49 RN‐hours per week (seven days with three shifts each) and 1.3 RN FTEs per year. The second largest estimate was found for HoNOS item 9 (‘problems with relationships’) in the simultaneous‐item model, with a 20% increase associated with 37 RN‐hours per week (1 RN FTEs per year). Across the single‐item models, weekly changes ranged from 16 to 30 RN‐hours (0.4–0.8 RN FTEs per year). Results for 10% and 30% increases are also presented in Table 3.

**TABLE 3:** Estimated RN staffing changes under varying HoNOS compositions.

	Total score model	Single‐item models						Simultaneous‐item model
Change in HoNOS	HoNOS Total Score	HoNOS 1	HoNOS 4	HoNOS 8	HoNOS 9	HoNOS 10	HoNOS 12	HoNOS 9
+ 10%: Week Hours	24.26	12.77	14.91	13.52	14.44	10.03	8.06	18.35
+ 10%: Year FTE	0.67	0.35	0.41	0.37	0.40	0.28	0.22	0.50
+ 20%: Week Hours	48.52	25.54	29.82	27.04	28.88	20.07	16.13	36.70
+ 20%: Year FTE	1.33	0.70	0.82	0.74	0.79	0.55	0.44	1.01
+ 30%: Week Hours	72.78	38.31	44.72	40.56	43.33	30.10	24.19	55.04
+ 30%: Year FTE	2.00	1.05	1.23	1.12	1.19	0.83	0.67	1.51

**FIGURE 2:**
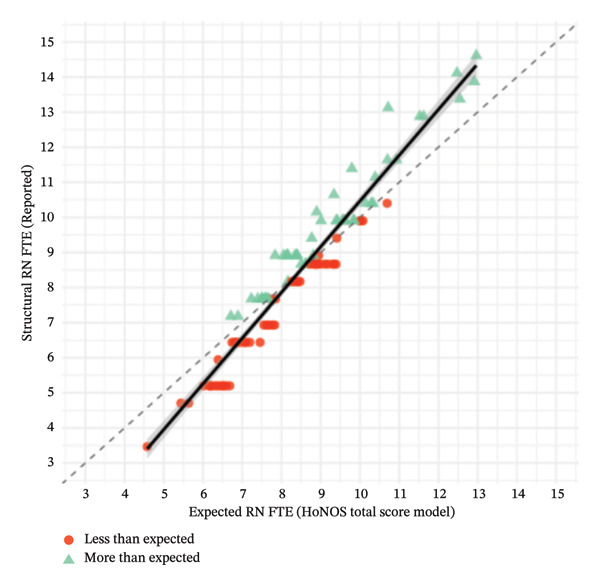
Comparison of structural and estimated RN FTEs per year by unit based on the total score model (modelling approach 1).

Based on the fitted models, we also calculated the RN FTEs predicted for each unit and compared these values with survey‐reported structural staffing. This comparison is illustrated in Figure 2, which shows, for each unit, whether reported staffing was above or below the model‐predicted value. Results are shown for the HoNOS total score model; results for the other models were similar (Supporting Figures 1 and 2). Overall, units with higher total RN FTEs more often appeared above model‐predicted values, whereas smaller units with fewer RN FTEs more often appeared below them.

## 4. Discussion

This study examined structural characteristics, patient mental health status and nurse staffing in Swiss psychiatric inpatient care. We found substantial variation in unit‐level characteristics, significant associations between HoNOS scores and RN staffing, and structural differences between small and large units. Based on the regression coefficients, a hypothetical 20% increase in the HoNOS total score was associated with higher structural staffing of more than one RN full‐time equivalent per year, underscoring that even moderate changes in patient acuity may be associated with meaningful shifts in RN staffing. At the same time, the predicted‐versus‐reported comparison showed a size‐related pattern, with smaller units more often falling below and larger units above model‐predicted values.

We observed marked variation across units in compulsory admissions, coercive measures, patient age, length of stay and HoNOS item scores. Similar heterogeneity has been reported internationally and is often linked to differences in diagnostic mix, ethnicity and socio‐economic deprivation in catchment areas [34–36]. In Switzerland, such a variation is likely further shaped by cantonal legislation and service delivery mandates across hospitals [37]. These findings underline the heterogeneity of psychiatric inpatient care and suggest that variables such as coercive measures may be relevant for future staffing models. At the same time, they highlight the difficulty of capturing staffing‐relevant patient need with a single standardised measure. Although HoNOS provides one of the few routinely available standardised indicators in this setting, it cannot fully represent the complexity of psychiatric inpatient populations. Local legal and organisational contexts therefore remain important when interpreting unit‐level differences.

Despite the marked variation in patient and unit characteristics, the basic staffing pattern was relatively similar across units. Median RN and LPN staffing levels in our sample were comparable to those reported in French‐speaking Switzerland, where two nurses per day and late shift were also found on psychiatric wards [38]. By including LPNs, our study captures an often overlooked but essential part of the workforce and thereby also reflects differences in educational preparation within nursing teams. The data represent structural or ‘typical’ staffing patterns across shifts and weekdays, rather than observed staffing at the level of single teams. This means that characteristics such as years of professional experience or the degree of team familiarity are not captured, even though research suggests that experience and established working relationships, alongside education, can shape how care is provided and experienced [39, 40].

Reporting on nurse staffing is challenging in psychiatry as in other inpatient settings. International studies often use patient‐to‐nurse ratios without distinguishing between shifts or facility types [41, 42]. Other approaches, such as ours, incorporate unit size and capacity utilisation to account for structural variation and actual occupancy. Each method highlights different dimensions of staffing and carries its own limitations. In mental health care, in particular, cross‐national comparisons are further complicated by the fact that care delivery systems differ widely between countries, shaping both how psychiatric care is organised and how staffing requirements are defined.

Using the HoNOS as an indicator for symptom severity, we modelled how changes in severity were associated with structural staffing. A 20% increase in the HoNOS total score corresponded to an additional 1.1 RN hours per shift, which equates to more than one RN full‐time equivalent over the course of a year. While the effect sizes may appear modest at the shift level, they may be operationally meaningful given the small baseline number of nurses per shift. Thus, even moderate differences in the average symptom severity of a unit’s patient population may correspond to meaningful differences in structural staffing levels. Our data do not allow conclusions about patient care demand on individual shifts, but the annualised magnitude of the association suggests that temporal fluctuations in patient composition may translate into periods in which routine staffing levels are less well matched to the unit’s current patient population, with potential implications for staffing planning, nurse workload and patient care.

In addition to the total score, several individual HoNOS items were significantly associated with RN staffing. The strongest associations were for item 9 (problems with relationships), item 4 (cognitive problems), item 1 (overactive/aggressive/disruptive/agitated behaviour), item 8 (other mental and behavioural problems), item 10 (problems with activities of daily living) and item 12 (problems with occupation and activities). These findings point to dimensions of patient demand that extend beyond symptom severity, highlighting the relevance of social functioning, cognitive impairment and relational difficulties for determining staffing needs.

Structural characteristics also contributed to explaining variation in RN staffing. Unit size showed a positive association across all models, reflecting the higher absolute number of staff in larger units. PSWs and LPN staffing were likewise consistently selected with negative associations, although only the effect of peer support reached statistical significance in some models. This pattern may indicate a partial substitution of RNs with other staff groups, a development that is debated in the literature given concerns about potential effects on care quality [8]. Length of stay was negatively associated with RN staffing in all models, suggesting that units with longer average stays tend to operate with lower structural RN staffing.

When each unit’s observed staffing was compared with the value predicted by the models, smaller units tended to fall below and larger units above their predicted levels. Because the models were fitted to reported structural staffing, their predicted values capture current practice, not required staffing, and the differences from observed values are essentially residuals rather than deviations from a desired standard. Within this large multicentre sample, smaller units consistently fell below and larger units above their predicted levels, yet neither deviation can be read as under‐ or over‐staffing without a standard of adequacy we do not have. Smaller units may require fewer staff or be relatively disadvantaged, just as larger units may require more or be comparatively well resourced. Clinically, however, evidence on the effects of unit size and crowding is limited and contradictory [43, 44]. Nevertheless, expert consensus holds that limiting unit size supports a positive ward atmosphere and reduces the risk of aggressive behaviour, a viewpoint reflected, for instance, in German guidance recommending a maximum of 18 beds per adult psychiatric unit Gemeinsamer Bundesausschuss [45, 46]. These perspectives highlight that staffing levels and unit structures need to be considered together: smaller units may better support therapeutic care but require sufficient resources to ensure safe and sustainable staffing.

A central contribution of this study is to demonstrate that routinely collected data can be linked to nurse staffing in psychiatric inpatient care. Both the HoNOS total score and selected single items were associated with RN staffing, showing that routinely assessed symptom severity and functional problems can serve as indicators of patient demand. The HoNOS has been widely used in research on patient outcomes, service utilisation and compulsory admissions [16], and our findings extend this evidence by highlighting its relevance for staffing.

Other approaches to capturing workload, such as situational assessments, may reflect short‐term variation more precisely [38, 47]. However, they require additional documentation effort, which reduces time for direct care. In the context of workforce shortages, limiting administrative burden is particularly important, as it may positively affect nurse satisfaction, recruitment and retention [48]. Routine data therefore offer a pragmatic alternative: while less granular, they are continuously collected and carry no extra burden for staff.

Finally, our study contributes to the broader debate on staffing tools. Although numerous instruments exist, evidence that they improve outcomes compared to expert judgement remains limited [49]. Rather than proposing a new tool, we emphasise the potential of existing routine data systems to support staffing decisions. Harnessing such data could help avoid workload peaks, improve continuity of care and provide organisations with information to guide strategic responses.

### 4.1. Strengths and Limitations

This multicentre study combined routinely collected patient data with survey data from unit managers; the high response rate contributed to a comprehensive dataset on psychiatric inpatient staffing in Switzerland. The use of multilevel models allowed us to account for the hierarchical structure of the data and yielded good model fit. Several limitations must be considered. Staffing information was collected through the unit‐manager survey between November 2023 and April 2024, whereas routine patient data covered discharges in 2022. The analysis therefore assumes that the typical staffing configuration reported by managers reflected relatively stable structural staffing patterns that were already in place during, or at least comparable to, the 2022 reporting period. This assumption is plausible for baseline staffing configurations, which are usually linked to unit size, service mandate and organisational planning rather than to daily fluctuations. However, changes in staffing models, vacancies, temporary closures, ward reorganisations or changes in patient mix between 2022 and 2023/2024 could not be captured. Patient data could only be attributed to the last unit of stay, which may have introduced some misclassification, although transfers between units are relatively uncommon in psychiatric care.

## 5. Conclusions

This study shows that routinely collected clinical data, particularly HoNOS scores, were associated with manager‐reported structural RN staffing in psychiatric inpatient care. Variation in patient composition, as reflected in HoNOS, is mirrored in current staffing, while differences between smaller and larger units indicate that staffing is shaped by structural factors in addition to patient acuity. These findings suggest that routine data may provide a pragmatic basis for more transparent strategic staffing discussions, particularly when interpreted alongside structural unit characteristics and local organisational context. Embedding routine data into staffing processes could help move resource allocation away from less systematic approaches and towards more transparent strategies that support sustainable workforce planning.

## Author Contributions

Michael Ketzer: writing–original draft, formal analysis, visualisation, project administration, conceptualisation and data curation. Beatrice Gehri: methodology, writing–review and editing and supervision. Christian G. Huber and André Nienaber: conceptualisation and writing–review and editing. Michael Simon: writing–review and editing, methodology and supervision.

## Funding

The Match^RN^ Psychiatry study was funded by the Participating Psychiatric Hospitals and by the Swiss Psychiatric Nursing Leaders’ Association (VPPS). Open access publishing facilitated by Universitat Basel, as part of the Wiley‐Universitat Basel agreement via the Consortium of Swiss Academic Libraries.

## Conflicts of Interest

The authors declare no conflicts of interest.

## Supporting Information

Additional supporting information can be found online in the Supporting Information section.

## Supporting information


**Supporting Information** Supporting Table 1. Regression estimates and confidence intervals for HoNOS items and total score across three modelling approaches, showing the full set of results. Supporting Figure 1. Comparison of structural and estimated registered nurse (RN) full‐time equivalents (FTEs) per year by unit based on the single‐item models (Modelling Approach 2). Supporting Figure 2. Comparison of structural and estimated registered nurse (RN) full‐time equivalents (FTEs) per year by unit based on the simultaneous‐item model (Modelling Approach 3). Supporting Table 2. Candidate predictors and bootstrap lasso selection outcome by modelling approach.

## Data Availability

The data that support the findings of this study are available on request from the corresponding author. The data are not publicly available due to privacy or ethical restrictions.
