# Supplementary material for: Nurse Staffing in Psychiatric Inpatient Care: A Multicentre Study Using Routine Patient and Structural Data
Source: J Nurs Manag. 2026 Jul 30;2026:5552920. doi: 10.1155/jonm/5552920 (PMC13420185; doi:10.1155/jonm/5552920)
Supplement: Supplementary file 1 — Supporting Information Supporting Table 1. Regression estimates and confidence intervals for HoNOS items and total score across three modelling approaches, showing the full set of results. Supporting Figure 1. Comparison of structural and estimated registered nurse (RN) full‐time equivalents (FTEs) per year by unit based on the single‐item models (Modelling Approach 2). Supporting Figure 2. Comparison of structural and estimated registered nurse (RN) full‐time equivalents (FTEs) per year by unit based on the simultaneous‐item model (Modelling Approach 3). Supporting Table 2. Candidate predictors and bootstrap lasso selection outcome by modelling approach. [file JONM-2026-5552920-s001.zip › Supplementary material.docx]

**Supplement**

|  | **Total score model** | **Single-item models** | | | | | | | | | | | | **Simultaneous-item model** |
| --- | --- | --- | --- | --- | --- | --- | --- | --- | --- | --- | --- | --- | --- | --- |
|  | **HoNOS Total Score** | **HoNOS 1** | **HoNOS 2** | **HoNOS 3** | **HoNOS 4** | **HoNOS 5** | **HoNOS 6** | **HoNOS 7** | **HoNOS 8** | **HoNOS 9** | **HoNOS 10** | **HoNOS 11** | **HoNOS 12** |  |
| **Intercept** | 12.86* [1.53, 24.19] | 14.73* [3.43, 26.03] | 14.71* [3.01, 26.42] | 16.17** [4.32, 28.02] | 19.96*** [8.21, 31.70] | 18.65** [6.52, 30.78] | 14.04* [2.36, 25.73] | 14.79* [3.35, 26.22] | 13.73* [2.44, 25.03] | 15.50** [4.48, 26.52] | 16.95** [5.61, 28.30] | 14.73* [3.21, 26.24] | 14.78* [3.39, 26.17] | 18.66** [7.39, 29.93] |
| **HoNOS Total Score (0-1 scaled)** | 11.55** [2.95, 20.15] |  |  |  |  |  |  |  |  |  |  |  |  |  |
| **HoNOS 1** |  | 6.08* [0.75, 11.41] |  |  |  |  |  |  |  |  |  |  |  |  |
| **HoNOS 2** |  |  | 1.29 [-7.52, 10.10] |  |  |  |  |  |  |  |  |  |  | -4.00 [-11.98, 3.99] |
| **HoNOS 3** |  |  |  | -1.89 [-5.77, 1.99] |  |  |  |  |  |  |  |  |  | -1.14 [-4.52, 2.25] |
| **HoNOS 4** |  |  |  |  | 7.10** [2.27, 11.93] |  |  |  |  |  |  |  |  | 2.51 [-3.33, 8.35] |
| **HoNOS 5** |  |  |  |  |  | 7.94+ [-0.17, 16.06] |  |  |  |  |  |  |  |  |
| **HoNOS 6** |  |  |  |  |  |  | 2.95 [-2.80, 8.71] |  |  |  |  |  |  |  |
| **HoNOS 7** |  |  |  |  |  |  |  | 3.94+ [-0.37, 8.25] |  |  |  |  |  |  |
| **HoNOS 8** |  |  |  |  |  |  |  |  | 6.44** [1.86, 11.02] |  |  |  |  | 0.53 [-5.50, 6.56] |
| **HoNOS 9** |  |  |  |  |  |  |  |  |  | 6.88** [2.78, 10.98] |  |  |  | 8.74* [0.65, 16.82] |
| **HoNOS 10** |  |  |  |  |  |  |  |  |  |  | 4.78** [1.15, 8.41] |  |  |  |
| **HoNOS 11** |  |  |  |  |  |  |  |  |  |  |  | 3.52 [-1.50, 8.53] |  | -4.36 [-11.45, 2.73] |
| **HoNOS 12** |  |  |  |  |  |  |  |  |  |  |  |  | 3.84* [0.07, 7.61] |  |
| **Weekend shift** | -3.73*** [-4.43, -3.03] | -3.73*** [-4.43, -3.03] | -3.72*** [-4.42, -3.02] | -3.72*** [-4.42, -3.02] | -3.73*** [-4.43, -3.03] | -3.73*** [-4.43, -3.03] | -3.73*** [-4.43, -3.03] | -3.73*** [-4.43, -3.02] | -3.73*** [-4.43, -3.03] | -3.74*** [-4.44, -3.04] | -3.74*** [-4.44, -3.04] | -3.73*** [-4.43, -3.03] | -3.73*** [-4.43, -3.03] | -3.73*** [-4.43, -3.03] |
| **Late shift** | -2.63*** [-3.49, -1.76] | -2.61*** [-3.48, -1.75] | -2.61*** [-3.47, -1.74] | -2.61*** [-3.47, -1.74] | -2.61*** [-3.48, -1.75] | -2.61*** [-3.48, -1.75] | -2.61*** [-3.47, -1.75] | -2.61*** [-3.47, -1.75] | -2.61*** [-3.48, -1.75] | -2.63*** [-3.49, -1.77] | -2.63*** [-3.49, -1.76] | -2.62*** [-3.48, -1.75] | -2.62*** [-3.49, -1.76] | -2.62*** [-3.48, -1.76] |
| **Night shift** | -8.63*** [-9.62, -7.64] | -8.59*** [-9.58, -7.60] | -8.57*** [-9.57, -7.58] | -8.57*** [-9.56, -7.58] | -8.60*** [-9.59, -7.61] | -8.59*** [-9.58, -7.60] | -8.59*** [-9.58, -7.60] | -8.59*** [-9.58, -7.60] | -8.59*** [-9.58, -7.60] | -8.64*** [-9.63, -7.65] | -8.63*** [-9.62, -7.64] | -8.60*** [-9.59, -7.61] | -8.62*** [-9.61, -7.63] | -8.61*** [-9.60, -7.62] |
| **Private ownership** | -0.42 [-2.53, 1.70] | -0.53 [-2.68, 1.61] | -0.66 [-2.87, 1.55] | -0.94 [-3.16, 1.28] | -0.47 [-2.58, 1.63] | -0.06 [-2.31, 2.19] | -0.64 [-2.81, 1.53] | -0.87 [-3.02, 1.28] | -0.49 [-2.60, 1.63] | -0.26 [-2.34, 1.82] | -0.33 [-2.46, 1.80] | -0.41 [-2.61, 1.79] | -0.52 [-2.67, 1.62] | -0.75 [-2.90, 1.39] |
| **Outpatient service** | -0.33 [-1.77, 1.11] | -0.16 [-1.64, 1.32] | -0.44 [-1.94, 1.06] | -0.60 [-2.11, 0.90] | -0.41 [-1.85, 1.03] | -0.31 [-1.79, 1.18] | -0.56 [-2.05, 0.93] | -0.44 [-1.90, 1.03] | -0.26 [-1.71, 1.19] | -0.29 [-1.71, 1.12] | -0.43 [-1.87, 1.01] | -0.42 [-1.90, 1.05] | -0.47 [-1.93, 0.99] | -0.40 [-1.79, 0.99] |
| **Licensed practical nurses** | -0.54 [-1.35, 0.26] | -0.49 [-1.29, 0.32] | -0.46 [-1.27, 0.35] | -0.46 [-1.27, 0.35] | -0.50 [-1.30, 0.31] | -0.49 [-1.30, 0.32] | -0.48 [-1.29, 0.33] | -0.48 [-1.29, 0.33] | -0.49 [-1.30, 0.32] | -0.56 [-1.36, 0.25] | -0.55 [-1.36, 0.26] | -0.51 [-1.32, 0.30] | -0.53 [-1.34, 0.28] | -0.52 [-1.32, 0.28] |
| **Peer support service** | -1.17 [-2.71, 0.36] | -1.65* [-3.16, -0.14] | -1.65* [-3.23, -0.07] | -1.82* [-3.37, -0.27] | -1.02 [-2.58, 0.53] | -1.17 [-2.79, 0.44] | -1.73* [-3.26, -0.19] | -1.41+ [-2.96, 0.14] | -1.44+ [-2.94, 0.07] | -1.16 [-2.66, 0.33] | -1.27 [-2.79, 0.26] | -1.53+ [-3.08, 0.02] | -1.38+ [-2.92, 0.17] | -1.19 [-2.74, 0.36] |
| **Unit always open** | 1.89+ [-0.05, 3.83] | 1.55 [-0.31, 3.42] | 1.41 [-0.67, 3.48] | 1.24 [-0.72, 3.20] | 1.44 [-0.66, 3.53] | 2.01+ [-0.23, 4.25] | 1.56 [-0.45, 3.58] | 1.24 [-0.69, 3.18] | 1.40 [-0.70, 3.50] | 1.75+ [-0.13, 3.62] | 1.83+ [-0.11, 3.77] | 1.77+ [-0.28, 3.82] | 1.74+ [-0.23, 3.72] | 1.14 [-0.87, 3.15] |
| **Capacity utilisation** | -1.48 [-17.13, 14.16] | 3.19 [-11.99, 18.38] | 4.28 [-11.32, 19.89] | 5.71 [-9.94, 21.36] | -3.14 [-18.99, 12.70] | -0.90 [-17.19, 15.38] | 4.17 [-11.30, 19.64] | 1.86 [-13.69, 17.41] | -0.51 [-15.95, 14.93] | -2.88 [-18.24, 12.48] | -1.85 [-17.62, 13.93] | 2.61 [-13.02, 18.23] | 0.89 [-14.73, 16.52] | -4.32 [-20.22, 11.58] |
| **Unit size (number of beds)** | 0.18** [0.04, 0.31] | 0.17* [0.03, 0.31] | 0.16* [0.02, 0.29] | 0.17* [0.03, 0.31] | 0.19** [0.06, 0.32] | 0.17* [0.03, 0.31] | 0.16* [0.03, 0.30] | 0.15* [0.01, 0.28] | 0.19** [0.06, 0.33] | 0.19** [0.06, 0.32] | 0.19** [0.05, 0.32] | 0.17* [0.03, 0.30] | 0.16* [0.03, 0.30] | 0.21** [0.07, 0.34] |
| **Age** | -0.01 [-0.07, 0.05] | -0.02 [-0.08, 0.04] | -0.01 [-0.08, 0.06] | -0.02 [-0.08, 0.04] | -0.04 [-0.10, 0.02] | -0.08+ [-0.17, 0.01] | -0.01 [-0.07, 0.05] | 0.01 [-0.06, 0.07] | 0.00 [-0.06, 0.06] | 0.02 [-0.04, 0.08] | -0.02 [-0.08, 0.03] | -0.02 [-0.08, 0.04] | -0.00 [-0.06, 0.06] |  |
| **Proportion of female patients** | 1.93 [-3.51, 7.38] | 1.49 [-3.99, 6.98] | 1.42 [-4.99, 7.83] | -0.29 [-7.45, 6.86] | 1.26 [-4.21, 6.73] | 2.23 [-3.36, 7.82] | 2.31 [-3.37, 7.98] | -0.39 [-6.46, 5.68] | -1.38 [-7.33, 4.57] | 0.73 [-4.66, 6.12] | 1.70 [-3.75, 7.16] | 2.09 [-3.51, 7.68] | 2.21 [-3.33, 7.74] |  |
| **Compulsory admission** |  |  |  |  | -2.56 [-8.59, 3.48] | 1.44 [-4.50, 7.38] |  |  | 2.05 [-3.79, 7.90] |  |  |  |  |  |
| **Coercive measures** | 5.41 [-2.14, 12.96] |  | 5.83 [-1.99, 13.64] | 4.83 [-3.24, 12.89] | 4.44 [-4.46, 13.34] | 4.67 [-4.45, 13.80] | 4.33 [-4.00, 12.66] | 7.71+ [-0.24, 15.65] | 3.86 [-5.12, 12.84] | 5.01 [-2.41, 12.43] | 5.35 [-2.21, 12.92] | 5.85 [-1.89, 13.58] | 6.51+ [-1.17, 14.20] | 2.97 [-4.84, 10.78] |
| **Length of stay** | -0.06+ [-0.12, 0.01] | -0.06+ [-0.13, 0.01] | -0.08* [-0.14, -0.01] | -0.09* [-0.16, -0.02] | -0.07* [-0.13, -0.00] | -0.07* [-0.14, -0.01] | -0.08* [-0.15, -0.01] | -0.08* [-0.14, -0.01] | -0.07* [-0.14, -0.00] | -0.06+ [-0.12, 0.01] | -0.06+ [-0.13, 0.00] | -0.07* [-0.14, -0.00] | -0.07* [-0.14, -0.00] | -0.07* [-0.14, -0.00] |
| **SD (Intercept unit_id)** | 2.90 | 2.96 | 3.04 | 3.02 | 2.89 | 2.98 | 3.02 | 2.97 | 2.90 | 2.82 | 2.90 | 3.00 | 2.96 | 2.79 |
| **SD (Observations)** | 4.42 | 4.42 | 4.43 | 4.43 | 4.42 | 4.42 | 4.42 | 4.42 | 4.42 | 4.42 | 4.42 | 4.42 | 4.42 | 4.42 |
| **Num.Obs.** | 636 | 636 | 636 | 636 | 636 | 636 | 636 | 636 | 636 | 636 | 636 | 636 | 636 | 636 |
| **R2 Marg.** | 0.390 | 0.381 | 0.373 | 0.375 | 0.393 | 0.381 | 0.376 | 0.381 | 0.391 | 0.399 | 0.389 | 0.378 | 0.383 | 0.406 |
| **R2 Cond.** | 0.573 | 0.572 | 0.574 | 0.574 | 0.574 | 0.575 | 0.574 | 0.574 | 0.574 | 0.572 | 0.573 | 0.574 | 0.573 | 0.575 |
| **AIC** | 3842.4 | 3850.0 | 3849.1 | 3849.9 | 3840.2 | 3843.6 | 3849.0 | 3847.4 | 3841.0 | 3840.3 | 3844.4 | 3848.4 | 3846.9 | 3821.1 |
| **BIC** | 3922.6 | 3925.7 | 3929.3 | 3930.1 | 3924.9 | 3928.2 | 3929.2 | 3927.6 | 3925.6 | 3920.5 | 3924.6 | 3928.6 | 3927.1 | 3914.6 |
| **ICC** | 0.3 | 0.3 | 0.3 | 0.3 | 0.3 | 0.3 | 0.3 | 0.3 | 0.3 | 0.3 | 0.3 | 0.3 | 0.3 | 0.3 |
| **RMSE** | 4.12 | 4.12 | 4.12 | 4.12 | 4.12 | 4.12 | 4.12 | 4.12 | 4.12 | 4.13 | 4.12 | 4.12 | 4.12 | 4.13 |
| **+ p < 0.1, * p < 0.05, ** p < 0.01, *** p < 0.001** | | | | | | | | | | | | | | |

Supplementary Table 1 Regression estimates and confidence intervals for HoNOS items and Total Score across three modelling approaches, full results

Supplementary Figure 1 Comparison of structural and estimated RN FTEs per year by unit, based on the single-item models (Modelling Approach 2)

Supplementary Figure 2 Comparison of structural and estimated RN FTEs per year by unit, based on the simultaneous-item model (Modelling Approach 3)

| **Candidate predictor** | **Variable domain** | **Total score model** | **Single-item models** | **Simultaneous-item model** |
| --- | --- | --- | --- | --- |
| **Weekend shift** | Shift indicator | Retained | Retained in 12/12 | Retained |
| **Late shift** | Shift indicator | Retained | Retained in 12/12 | Retained |
| **Night shift** | Shift indicator | Retained | Retained in 12/12 | Retained |
| **Private ownership** | Hospital characteristic | Retained | Retained in 12/12 | Retained |
| **Outpatient service** | Unit characteristic | Retained | Retained in 12/12 | Retained |
| **LPN staffing** | Staffing characteristic | Retained | Retained in 12/12 | Retained |
| **Peer support workers** | Unit characteristic | Retained | Retained in 12/12 | Retained |
| **Unit always open** | Unit characteristic | Retained | Retained in 12/12 | Retained |
| **Capacity utilisation** | Unit characteristic | Retained | Retained in 12/12 | Retained |
| **Unit size** | Unit characteristic | Retained | Retained in 12/12 | Retained |
| **Mean patient age** | Patient composition | Retained | Retained in 12/12 | Excluded |
| **Proportion female patients** | Patient composition | Retained | Retained in 12/12 | Excluded |
| **Compulsory admission** | Patient composition | Excluded | Retained in 3/12 | Excluded |
| **Coercive measures** | Patient composition | Retained | Retained in 11/12 | Retained |
| **Length of stay** | Patient composition | Retained | Retained in 12/12 | Retained |
| **HoNOS total score** | Patient acuity | Retained | Not applicable | Not applicable |
| **HoNOS item 1** | Patient acuity | Not applicable | Retained in HoNOS 1 model | Excluded |
| **HoNOS item 2** | Patient acuity | Not applicable | Retained in HoNOS 2 model | Retained |
| **HoNOS item 3** | Patient acuity | Not applicable | Retained in HoNOS 3 model | Retained |
| **HoNOS item 4** | Patient acuity | Not applicable | Retained in HoNOS 4 model | Retained |
| **HoNOS item 5** | Patient acuity | Not applicable | Retained in HoNOS 5 model | Excluded |
| **HoNOS item 6** | Patient acuity | Not applicable | Retained in HoNOS 6 model | Excluded |
| **HoNOS item 7** | Patient acuity | Not applicable | Retained in HoNOS 7 model | Excluded |
| **HoNOS item 8** | Patient acuity | Not applicable | Retained in HoNOS 8 model | Retained |
| **HoNOS item 9** | Patient acuity | Not applicable | Retained in HoNOS 9 model | Retained |
| **HoNOS item 10** | Patient acuity | Not applicable | Retained in HoNOS 10 model | Excluded |
| **HoNOS item 11** | Patient acuity | Not applicable | Retained in HoNOS 11 model | Retained |
| **HoNOS item 12** | Patient acuity | Not applicable | Retained in HoNOS 12 model | Excluded |

Supplementary Table 2 Candidate predictors and bootstrap Lasso selection outcome by modelling approach
